# A Novel Method with Stacking Learning of Data-Driven Soft Sensors for Mud Concentration in a Cutter Suction Dredger

**DOI:** 10.3390/s20216075

**Published:** 2020-10-26

**Authors:** Bin Wang, Shi-dong Fan, Pan Jiang, Han-hua Zhu, Ting Xiong, Wei Wei, Zhen-long Fang

**Affiliations:** School of Energy and Power Engineering, Wuhan University of Technology, Wuhan 430063, China; wang_bin@whut.edu.cn (B.W.); river@whut.edu.cn (P.J.); hhzhu@whut.edu.cn (H.-h.Z.); cxj_xt@whut.edu.cn (T.X.); wei_wei@whut.edu.cn (W.W.); zl_fang@whut.edu.cn (Z.-l.F.)

**Keywords:** mud concentration, data mining, soft sensor, machine learning, dredger

## Abstract

The dredger construction environment is harsh, and the mud concentration meter can be damaged from time to time. To ensure that the dredger can continue construction operations when the mud concentration meter is damaged, the development of a dredger with advantages of low price and simple operation that can be used in emergency situations is essential. The characteristic spare mud concentration meter is particularly critical. In this study, a data-driven soft sensor method is proposed that can predict the mud concentration in real time and can mitigate current marine mud concentration meter malfunctions, which affects continuous construction. This sensor can also replace the mud concentration meter when the construction is stable, thereby extending its service life. The method is applied to two actual construction cases, and the results show that the stacking generalization (SG) model has a good prediction effect in the two cases, and its goodness of fit *R*^2^ values are as high as 0.9774 and 0.9919, indicating that this method can successfully detect the mud concentration.

## 1. Introduction

Cutter suction dredgers are very widely used in dredging projects because they have a wide range of digging depths, and are well adapted to the soil quality. This equipment can complete the dredging, conveying, discharge, and mud treatment processes at one time and can continue construction work, so the construction efficiency is higher [1,2]. The dredger’s output during the construction operation is obtained by the product of the mud concentration and the flow rate. Therefore, the determination of the mud concentration is essential during actual construction. This property affects the calculation of the output and affects the construction personnel’s judgement of the construction status. To increase the dredging output, construction workers will often try to increase the mud concentration without system failure. However, the adjustment of mud concentration is limited by conditions such as the mud pump’s power, the flow of the pump, and the suction vacuum. In actual construction, the mud concentration often does not exceed 40%. If the concentration is too high, then it will increase the loss along the pipeline, increase the wear of the equipment, and cause cavitation and pipe blockage [3,4].

During actual construction, the construction personnel will control a series of instructions, such as the reamer speed, traverse speed, and pile-in distance, according to the mud concentration, so the mud concentration is an extremely important measurement in a dredging project [5]. To complete the construction task efficiently, safely, and economically, the real-time online and reliable measurement of the mud concentration is critical. However, due to the harsh environment of the dredging site, the failure of the mud concentration meter occurs from time to time. To ensure the normal progress of the construction, two different methods are used. The concentration measurement method is extremely critical for measuring the mud concentration. When the commonly used mud concentration meter fails, the timely supplementation of the backup mud concentration meter is an important guarantee for the continuous and efficient operation of the dredger. Besides, the backup mud concentration meter should have the characteristics of low price and convenient operation. At present, the commonly used continuous online instruments of dredgers for measuring the mud concentration include γ ray densitometers, ultrasonic densitometers, and inverted U-shaped densitometers. The γ-ray densitometers and ultrasonic densitometers are expensive. When two measuring instruments are installed on a ship at the same time, the cost is higher, resulting in a waste of resources. Although the price of the inverted U-shaped densitometer is relatively cheap, it is tremendous in size and inconvenient to set up, limiting its application in engineering [6].

In recent years, many researchers have used electrical tomography (ET) technology to measure the mud concentration in pipelines, which includes two main methods: electrical capacitance tomography (ECT) and electrical resistance tomography (ERT) [7,8,9]. The ECT system is mainly composed of three parts: capacitance sensor, capacitance acquisition unit, and image reconstruction system. The capacitance sensor is located on the periphery of the insulated pipeline. When the distribution of the material in the pipeline changes, the capacitance between the plates of the capacitance sensor will also change accordingly. The capacitance value between the plates is transmitted to the image reconstruction system through the capacitor acquisition and conversion circuit, and the image reconstruction system processes the data through a particular algorithm to obtain an image of the medium distribution in the pipeline. However, this method has a problem that is difficult to overcome in actual use. Specifically, the current dredging environment is complex, with many influencing factors. Noisy signals will affect the accuracy of the capacitance value. The current hardware conditions have difficulty overcoming the interference of external signals, so the application of ECT technology to the measurement of the mud concentration the actual construction is still very difficult. Alternatively, the ERT method uses current injection and voltage measurement methods. To find the target imaging area, when the other electrodes are in a floating state, an alternating current is applied to a pair of electrodes in sequence, and the resistance between the electrodes is measured, which continues until a complete set of data is obtained. Finally, an appropriate algorithm is used to reconstruct the image of the object or material distribution based on the measurement data. There are two problems in the practice of ERT: (1) The sensitive field established by the ERT sensor is three-dimensional, and its spatial distribution is nonuniform. Therefore, using an ideal two-dimensional field to approximate the three-dimensional field causes the measurement data on the sensitive electrode to be affected by the distribution of the medium in a specific spatial range, which in turn causes the reconstructed image to contain complex information. (2) The object of the ERT system to measure two-phase flow is generally a high-speed changing fluid, which has higher requirements for the sampling speed of the hardware.

It is a major research focus in the field of dredging to develop a mud concentration measurement method with the article advantages of low-cost, simple maintenance, and high-precision. To solve the measurement problem of certain variables in industrial production, the development of soft sensors and the resulting soft sensor technology are an important direction for the research and development of detection and process control [10,11,12]. There are two most commonly used methods for building soft sensor models, namely, mechanism modelling and data-driven modelling.

Soft sensor modelling based on mechanism analysis mainly uses the principles of chemical reaction kinetics, material balance, energy balance, etc. through the mechanism analysis of the process object to find the relationship between the unmeasured dominant variable and the measurable auxiliary variable (establish the mechanism model) to realize the soft sensor of a certain parameter. For a process with a precise process mechanism, this method can construct a soft instrument with good performance. However, it is not easy to establish a suitable mechanism model for complex industrial processes, with insufficient mechanism research, that are not fully understood. At this time, this method needs to be combined with other parameter estimation methods to construct a soft instrument. This soft-sensing modelling method is a commonly used method in engineering and is characterized by simplicity, with a transparent engineering background, which is convenient for practical applications. However, the application effect depends on the understanding of the process mechanism because this soft-sensing method is based on the process. Based on a deep understanding of the process mechanism, modelling is more difficult [13]. Wang et al. [14] using simulation modelling technology, successfully predicted the mud concentration of a dredger suction system, and the simulation model was verified by the dredging test bench. However, the comparison results showed that the accuracy of the mechanism model was not high but roughly predicted the changing trend of the mud concentration. Tang et al. [15] build a self-dredging operation system, achieving the online optimization and fault diagnosis of dredging operations. However, the system requires high accuracy and reliability of the sensor, which is not easy to implement in actual construction.

To break through the limitation of mechanism modelling, data-driven modelling methods have been developed in recent decades. These data-driven methods can be used to fit the easily measured process data and are applicable to most processes without requiring a deep understanding of their mechanisms [16]. Therefore, a large number of scholars have carried out a series of studies and applications on data-driven modelling methods. Among them, the most widely used linear method is a multiple statistical regression, such as Gaussian process regression (GPR) and partial least squares regression [17,18,19]. Because these methods are simple, they have strong practicability, but they are also prone to errors when dealing with complex data, especially data with impurities. Because most practical applications in production and life are nonlinear processes, the use of nonlinear models to deal with nonlinear processes has been widely recognized, such as artificial neural networks (ANNs), neuro-fuzzy (NF) systems, support vector machines (SVM), and principal component analysis (PCA) methods [20,21,22,23,24]. Although all of these nonlinear modelling methods solve the problems related to the nonlinear features, they all have issues such as model overfitting, complex multivariable system training models, and the insufficient processing of multistate behavior. Sometimes mixed models are used to improve the performance of each given category (linear and nonlinear) [25].

With the development of artificial intelligence technologies, such as machine learning and big data, soft-sensing technologies based on various new algorithms have been widely used in the biological, environmental, and chemical industries [26,27,28,29]. Deepak et al. [30] used the Bayesian regularized neural network to develop a local particulate matter (PM2.5) air quality early warning system. The core of the system is a soft sensor method constructed using the forward feature selection method of the Bayesian regularized neural network, and the air data are verified. The result shows that *R^2^* is as high as 0.95, and the prediction effect is significant.

Because of the particularity and complexity of the field of dredging, there are relatively fewer studies on the use of machine learning technology for soft sensors to measure the mud concentration. Bi et al. [31] started from the measurement principle of the elbow flow meter, the complex nonlinear relationship between the mud concentration and its influencing factors was derived, and a soft sensor of the mud concentration based on the radial basis function neural network was established by using the nonlinear mapping ability of the artificial neural network model. However, in the increasingly complex construction environment, the measurement accuracy of the model has become increasingly satisfactory.

In this research study, a new soft sensor method is proposed to predict the mud concentration based on data mining. First, 70% of the historical construction data in dredger are randomly selected as the training set, which is used to train the machine learning model. The remaining 30% of the data are utilized as the test set. Second, data pre-processing was performed on the training data and recursive feature elimination (RFE) was used to extract the features. Moreover, five machine learning models were used to learn and train the processed data to determine the parameters of each model. Then, the mud concentration was preliminarily predicted. Finally, the model with the best prediction effect was selected, and the stacking method was used to ensemble and generalize the multiple models. Through the verification of two actual cases, the final experimental results showed that the prediction of the stacking generalization (SG) model effect was obvious.

## 2. Analysis and Methods

**Figure 1:**
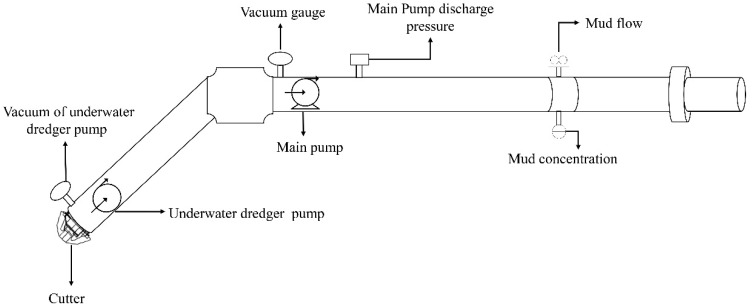
Main testing equipment of the mud pipeline transportation system.

The main data to be monitored by the mud transportation system include the pipeline mud concentration, mud flow, inlet and outlet pressures of various mud pumps, etc. The measurement of the mud pipeline concentration is mainly carried out on a γ-ray densitometer, which is widely used. The mud flow rate is mainly measured by the principle of electric measurement and induction. The pressure of each level of the mud pump is obtained by a pressure gauge. Figure 1 shows the main testing equipment of the mud pipeline transportation system.

During the process of the mud transportation, due to the harsh construction environment of the dredging site, the mud concentration meter is damaged from time to time, causing the dredger to be unable to perform normal continuous construction. Therefore, it is of great significance to configure a spare mud concentration meter on the existing dredger. However, the current marine γ mud concentration meter is expensive, and the configuration of two on the ship would be a great waste of resources, so the development of a low-cost, easy-to-operate mud concentration meter is imminent.

To mitigate the current industry constraints, in this paper, a new way of thinking about the soft sensor method to measure the mud concentration in a cutter suction dredger is proposed. First, a machine learning algorithm was used to mine and analyze the historical construction data of the dredger and determine the most important feature that affects the mud concentration in the corresponding algorithm. Second, the hyper-parameters for the dredger construction algorithm were determined by training and learning the algorithm model using the historical data of the selected features, and then the selected features were used to predict the mud concentration during the construction of the dredger, which could complement the mud concentration meter currently used in construction.

**Figure 2:**
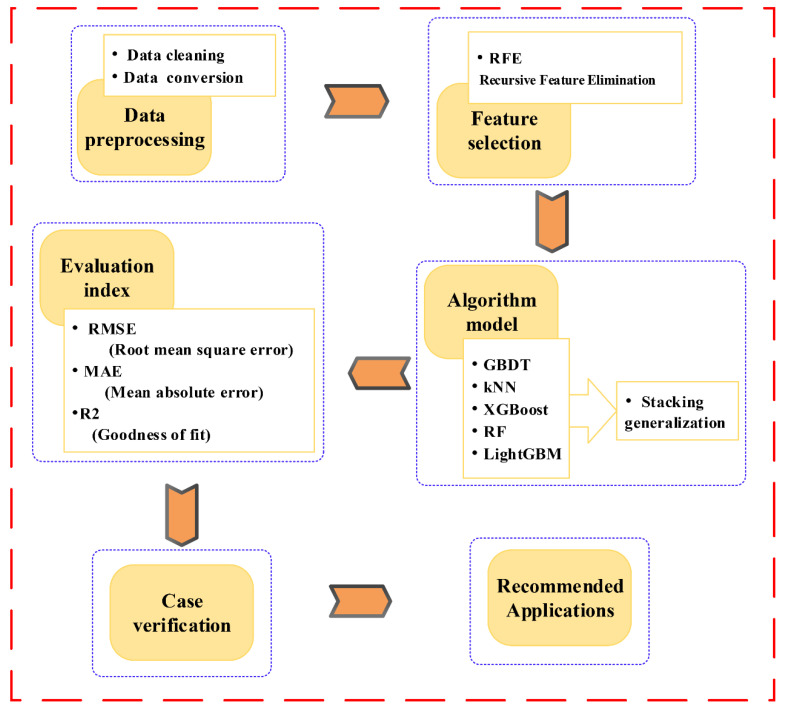
Technical framework of the article.

Figure 2 shows the proposed method framework:

### 2.1. Data Preprocessing

#### 2.1.1. Data Cleaning

Before the feature selection was performed, the construction status data with a mud concentration value of 0 were removed because the mud data were 0, which means that the dredger was not under construction. If these data are used for data mining, then the prediction accuracy will be affected.

The mud density, water density, and soil density data were removed because the mud density was calculated by the mud concentration data in the actual construction of the dredger, so it cannot be used to predict the mud concentration [32]. The conversion relationship was as Equation (1).

(1) $$C=\frac{\rho_{mud}-\rho_{water}}{\rho_{soil}-\rho_{water}}$$

where *C* is the mud concentration (*%*),$\rho_{mud}$ is the mud density (kg/m^3^), $\rho_{water}$ is the water density (kg/m^3^), and $\rho_{soil}$ is the soil density (kg/m^3^).

#### 2.1.2. Data Conversion

The feature vectors in the training data set have different units and orders of magnitude, which are very different from each other. If the original feature vector values are directly used for analysis, the role of high-value features in the comprehensive analysis will be strengthened, and low-value features will be weakened. Therefore, it is necessary to normalize the original feature data to ensure that all features are in the same dimension.

Normalized vector:

(2) $$\tilde{x}=\frac{x}{{\left\|x\right\|}_{2}}$$

Euclidean ($\ell_{2}$) norm:

(3) $${\left\|x\right\|}_{2}=\sqrt{x_{1}^{2}+x_{2}^{2}+\cdots +x_{m}^{2}}$$

After the normalization of the *L*_2_ norm, the feature vector data of each dimension are distributed on a spherical surface with a radius of 1, thereby eliminating the influence of the data unit on the prediction result. For linear models, the target value of the normal distribution is required to play the maximum role, so logarithmic conversion of the target value is performed, and then the least square method is used to smooth other characteristic data to approximate the normal distribution [33,34].

### 2.2. Feature Selection with RFE

This paper used the RFE feature selection method to select features from the dredger data. Nine feature vectors were finally selected, which can take into account calculation speed and accuracy at the same time [35]. The goal of RFE is to select features by recursively considering increasingly smaller sets of features. Support vector regression (SVR) as an external estimator was given that assigns weights to features [36]. First, the estimator is trained on the initial set of features, and the importance of each feature is obtained either through a “coefficient” attribute or through a feature “importance” attribute. Then, the least important features are pruned from the current set of features. This procedure is recursively repeated on the pruned set until the desired number of features to select is eventually reached.

### 2.3. Stacking Generalization 

#### 2.3.1. Stacking Generalization (SG)

SG is an ensemble method that allows the combination of several different algorithms into one: this method has been widely used since it was proposed in the 1990s [37,38,39]. This research integrates several classic machine learning methods brought into the SG model through the stacking method. The SG Algorithm 1 is as follows:
**Algorithm 1.** Stacking algorithm.**Inputs:** Training sets $D=\{(x_{1},y_{1}),(x_{2},y_{2}),\cdots ,(x_{m},y_{m})\}$
      Base model ${ℑ}_{1},{ℑ}_{2},\cdots ,{ℑ}_{T}$
      Meta-model ℑ
**Process:**
  1: **for**
t=1,2,…,T **do**
  2:   $h_{t}={ℑ}_{t}(D)$
  3: **end for**
  4: $D^{\prime }=\emptyset$
  5: **for**
i=1,2,…,m **do**
  6:    **for**
t=1,2,…,T **do**
  7:    $z_{it}=h_{t}(x_{i})$
  8:    **end for**
  9:  $D^{\prime }=D^{\prime }\cup ((z_{i1},z_{i2},\ldots ,z_{iT}),y_{i})$
  10: **end for**
  11:  $h^{\prime }=ℑ(D^{\prime })$
**Outputs**: $H(x)=h^{\prime }(h_{1}(x),h_{2}(x),\ldots ,h_{T}(x))$


In the training phase, the training set of the meta-model ℑ is generated by the base model learner ${ℑ}_{T}$, so the training set of the base model cannot be used directly to produce the training set of the meta-model, which may easily cause overfitting. Therefore, this paper adopts a five-fold cross-validation method, using unused samples of the base model to produce the training set of the meta-model; that is, the initial training set *D* is randomly divided into *k* sets $D_{1},D_{2},\ldots ,D_{k}$ of similar size, let $D_{j}$ and ${\bar{D}}_{j}=D\backslash D_{j}$ denote the test set and training set of the *j* fold, respectively. Given *T* base models, the base model $h_{t}^{\left(j\right)}$ is obtained by using the *t* base model on ${\bar{D}}_{j}$. For each sample $x_{i}$ in $D_{j}$, let $z_{it}=h_{{}_{t}}^{\left(j\right)}(x_{i})$, then the meta-model training set generated by $x_{i}$ is $z_{i}=(z_{i1};z_{i2};\ldots ;z_{iT})$, and the target part is $y_{i}$. After five-fold cross-validation, a new training set $D^{\prime }={\left\{(z_{i},y_{i})\right\}}_{i=1}^{m}$ generated from *T* base models can be obtained, and then $D^{\prime }$ is used to train the meta-model.

**Figure 3:**
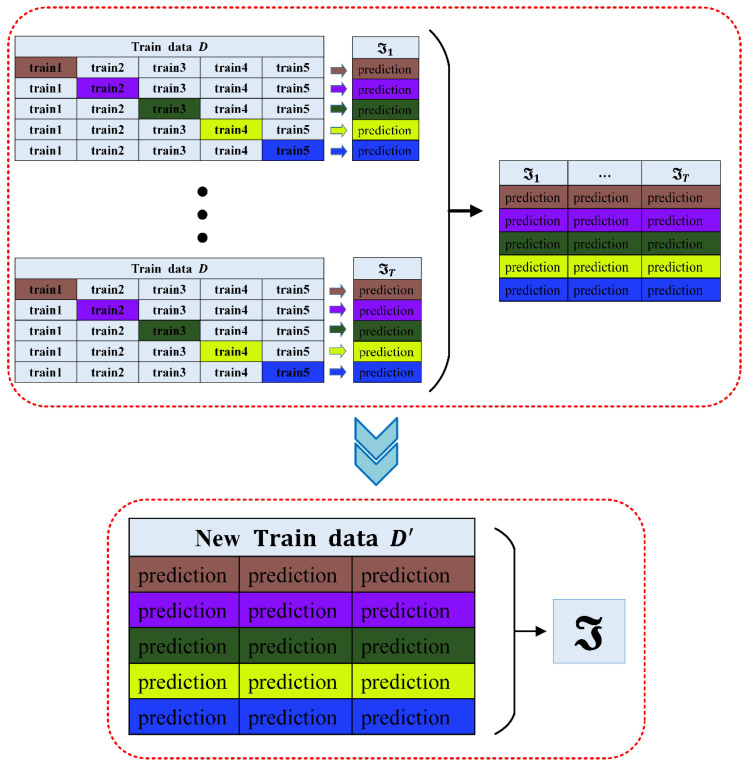
Stacking generalization (SG) schematic diagram.

The schematic diagram of the SG algorithm is shown in Figure 3:

#### 2.3.2. Gradient Boosting Decision Tree (GBDT)

Freidman [40] proposed the GBDT algorithm using the approximate method of the fastest descent method, which key is to use the value of the negative gradient of the loss function in the current model as the approximate value of the residual in the regression problem lifting tree Algorithm 2 to fit a regression tree.
**Algorithm 2.** Gradient boosting decision tree (GBDT) algorithm.**Inputs**: Training data set: $\begin{array}{cc} T=\{(x_{1}{,y}_{1}),(x_{2}{,y}_{2}),\cdots ,(x_{n}{,y}_{n})\} & \begin{array}{ccc} , & x_{i}\in \chi \subseteq R^{n},y_{i}\in ϒ\subseteq R & ; \end{array} \end{array}$     Loss function $L(y,f(x))=\frac{1}{2}{\left(y-f(x)\right)}^{2}$ (1) Initialize:
     $f_{0}(x)=argmin_{\gamma }\sum_{i=1}^{N}L(y_{i},\gamma )$ (2) **For** m = 1, 2, …, *M*
(a) **For**
*i* = 1, 2, …, *N*, compute
     $r_{im}=-{\left[\frac{\partial L(y_{i},f(x_{i}))}{\partial f(x_{i})}\right]}_{f=f_{m-1}}$
(b) Fit a regression tree to the targets $r_{\mathrm{im}}$ giving terminal regions *R*_jm_, *j* = 1, 2,…, *J*_m_.
(c) **For**
*j* = 1, 2, …, *J*_m_ compute
     $\gamma_{\mathrm{jm}}=\arg\underset{\gamma }{\min}\sum_{x_{i}\in R_{jm}}L(y_{i},f_{m-1}(x_{i})+\gamma )$
(d) **Update**
$f_{m}(x)=f_{m-1}(x)+\sum_{j=1}^{j_{m}}\gamma_{jm}I(x\in R_{jm})$
**Outputs:**
$\hat{f}(x)=f_{M}(x)$


#### 2.3.3. K-Nearest Neighbor (KNN)

KNN Algorithm 3 is a commonly used supervised learning method [41], in which a sample is most similar to the k samples in the data set. If most of these k samples belong to a certain category, then the sample also belongs to this category.
**Algorithm3.** K-nearest neighbor (KNN) algorithm.**Input**: Label data set $D_{l}={\left\{x_{i},y_{i}\right\}}_{i=1}^{l}$   Pseudocode labeled unlabeled data set $D_{u}={\left\{x_{j}\right\}}_{j=1}^{u}$   The parameter *K* in the KNN algorithm
**Process**
1. **for**
*j* = 1: *u*
2.   **for**
*i* = 1: *l*
3.    Calculate the Euclidean distance $S_{\mathrm{ji}}$ between $\left\{x_{j}\right\}$ and $\left\{x_{i}\right\}$
4.   **end**
5.   Sort the labelled data set $D_{l}$ in ascending order according to the distance $S_{\mathrm{ji}}$
6.   Select the first *K* data with tags, record their distance and tag information $\left\{s_{1},y_{1}\},\cdots ,\{s_{K}{,y}_{K}\right\}$
7.   **for**
*k* = 1: *K*
8.    $w_{k}=\exp({-s}_{k}^{2}/2)$
9.   **end**
   $y_{i}=\frac{\sum_{k=1}^{K}w_{k}y_{k}}{\sum_{k}^{K}w_{k}}$
11. **end**
12.   Get pseudo-labelled data set $D_{p}{=\{x}_{j}{,y}_{j}{\}}_{j=1}^{u}$
**Outputs:**
$D_{\mathrm{knn}}{=D}_{l}\cup D_{p}$


The advantages of the KNN algorithm are as follows: simple and effective, low retraining cost, low algorithm complexity, suitable for cross-samples of class domains, suitable for automatic classification of large samples. The disadvantages of the KNN algorithm are as follows: lazy learning, category classification is not standardized, output interpretability is not strong, uneven and highly computationally intensive.

#### 2.3.4. Extreme Gradient Boosting (XGBoost)

XGBoost is essentially an improvement to the GBDT algorithm [42], which has the advantages of fast speed, good effect, can handle large-scale data, supports multiple computer languages, supports custom loss functions, etc. The Algorithm 4 pseudo code is as follows:
**Algorithm 4.** Extreme gradient boosting (XGBoost) algorithm.**Input**: *I*, instance set of current node
**Input**: *d*, feature dimension
Gain ← 0
$G\leftarrow \sum_{i\in I}g_{i},H\leftarrow \sum_{i\in I}h_{i}$
**for**
*k* = 1 to *m*
**do**
    $G_{L}\leftarrow 0,H_{L}\leftarrow 0$
   **for**
*j* in sorted (*I*, by $x_{\mathrm{jk}}$) **do**
      $G_{L}\leftarrow G_{L}+g_{j},H_{L}\leftarrow H_{L}+h_{j}$
      $G_{R}\leftarrow G-G_{L},H_{R}\leftarrow H-H_{L}$
      $score\leftarrow max(score,\frac{G_{L}^{2}}{H_{L}+\lambda }+\frac{G_{R}^{2}}{H_{R}+\lambda }‒\frac{G^{2}}{H+\lambda })$
   **end**
**end**
**Output**: Split with max score

#### 2.3.5. Random Forest (RF)

Random forest (RF) is an important bagging-based ensemble learning method [43]. RF has many advantages; it has a very high accuracy rate and a good anti-noise ability, and the introduction of randomness makes the RF algorithm not easy to over fit. This method can handle very high-dimensional data without feature selection; it can handle both discrete data and continuous data and can easily realize parallelization. Additionally, the data set does not need to be standardized, the training speed is fast, and the importance of variables can be ranked. The RF method has disadvantages. For example, when there are many decision trees in the RF, the space and time required for training will be larger.

#### 2.3.6. Light Gradient Boosting Machine (LightGBM)

The LightGBM is a gradient boosting framework with fast, distributed, high-performance based on decision tree algorithm, which uses the histogram algorithm, dividing the continuous floating point features into k discrete values, displaying the cumulative statistics for each discrete value in the training set by the histogram with a width of k [44]. 

### 2.4. Evaluation Index

To quantitatively compare the prediction accuracy of each model, three evaluation indexes, including the root mean square error (RMSE), mean absolute error (MAE), and coefficient of determination (*R*^2^), are introduced.

(1) Root mean square error (RMSE)

(4) $$RMSE=\sqrt{\frac{1}{n}\sum_{i=1}^{n}{\left({\hat{y}}_{i}-y_{i}\right)}^{2}}$$

(2) Mean absolute error (MAE)

(5) $$MAE=\frac{1}{n}\sum_{i=1}^{n}\left|{\hat{y}}_{i}-y_{i}\right|$$

(3) Goodness of fit (*R*^2^)

(6) $$R^{2}=1-\frac{\sum_{i=1}^{n}{\left({\hat{y}}_{i}-y_{i}\right)}^{2}}{\sum_{i=1}^{n}{\left({\bar{y}}_{i}-y_{i}\right)}^{2}}$$

The MAE represents the average value of the absolute error between the predicted value and the true value, which can reflect the true situation of the predicted error. The RMSE represents the square root of the average of the squared difference between the predicted value and the true value. The smaller the value represented by MAE and RMSE, the better the performance of algorithm prediction. However, the RMSE and MAE have a drawback in evaluating the models, which cannot accurately evaluate indicators of different dimensions. So we used $R^{2}$ as an evaluation indicator, which can accurately evaluate features of different dimensions. The closer the $R^{2}$ score is to 1, the better the goodness of fit of the model is, and $R^{2}\in [0,1]$.

## 3. Case Study

To verify the mud concentration soft sensor method proposed in this paper, the method was applied to two different actual construction cases. Case 1 is the construction data of the “Chang Lion 10” cutter suction dredger on a certain day, and case 2 is the actual construction data of the “Chang Lion 12” cutter suction dredger. In every case, 70% of the data are randomly selected as the training set, which is used to train the machine learning model. The remaining 30% of the data are utilized as the test set.

### 3.1. Case 1

#### 3.1.1. Method

First, case 1 conducts a preliminary analysis of the construction data, which are the construction data from the “Chang Lion 10” cutter suction dredger in block 3 of Lianyungang Port, Jiangsu Province, China, on 23 June 2018. Second, 70% of the data are randomly selected as the training set, and the remaining data are selected as the test set. The construction data include 29,160 data sets (rows) with 239 dimensions (columns) from 8:27–15:25 Beijing time (BJT) on the studied day. The monitoring data structure of “Chang Lion 10” is shown in Table 1.

**Figure 4:**
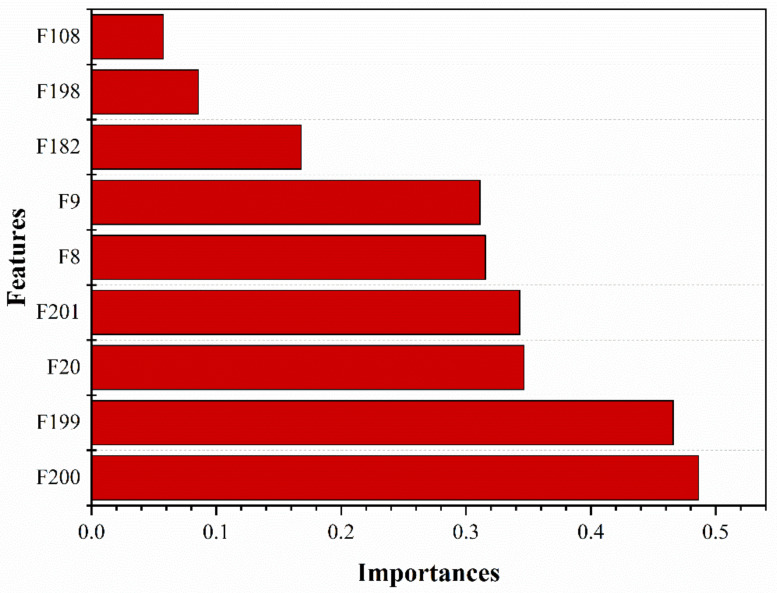
Feature importance ranking.

According to the method framework proposed in this paper, the original data were preprocessed first, and then RFE was used to select the features of the data. Nine feature columns that contributed the most to the mud concentration prediction were selected. The feature importance distribution is shown in Figure 4.

In Figure 4, where F200 is the 2 main pump discharge pressure, F199 is the 1 main pump discharge pressure, F20 is the flow rate, F201 is the vacuum of the underwater dredger pump, F8 is the ladder angle, F9 is the cutter depth, and F182 is the carriage stroke. F198 is the underwater dredger pump discharge pressure, and F108 is the power of the cutter.

The parameters of each algorithm model were selected by using a five-fold grid search cross-validation on the training data set and shown in Table 2.

#### 3.1.2. Evaluation

The performance of each algorithm model was compared and analyzed through three evaluation indicators. The specific evaluation indicators are shown in Table 3.

According to the single model comparison in Table 3, XGBoost is the best preforming model in terms of *R^2^*, MAE, and RMSE and the goodness of fit value of KNN was significantly smaller than the value of other algorithms.

The SG models constructed based on different single models are shown in Table 4. Close inspection of Table 4 reveals that the *R^2^* of SG model 4, with KNN, LightGBM, RF, and XGBoost as the base model and GBDT as the meta model, is the highest among all algorithm models, and MAE and RMSE are also the smallest, indicating that the SG model 4 can accomplish the task of accurately predicting mud concentration.

**Figure 5:**
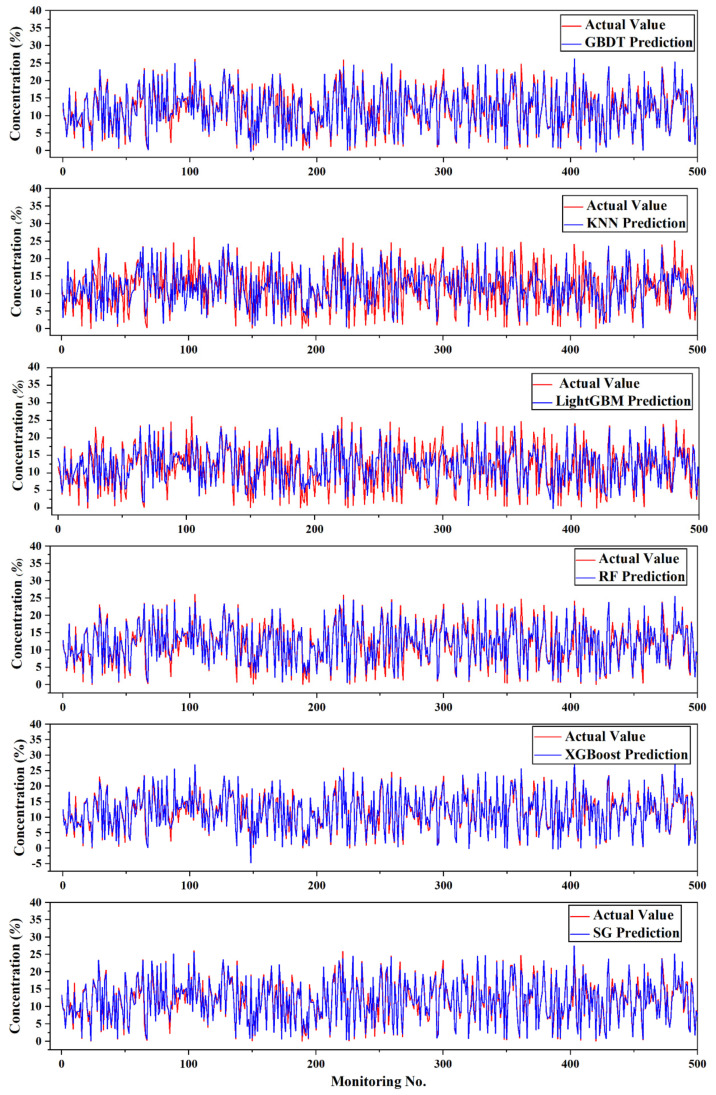
Error comparison.

To visually show the degree of deviation of the mud concentration prediction of each algorithm, 500 sets of test data were selected as the comparison analysis diagram between the predicted value and the true value, as shown in Figure 5. It can be seen more intuitively from Figure 5 that the predicted value of the SG algorithm was that the true value of the mud concentration had a higher degree of fit and a smaller prediction error.

### 3.2. Case 2

#### 3.2.1. Method

Case 2 first conducts a preliminary analysis of the construction data, which were the construction data from the “Chang Lion 12” cutter suction dredger in Nanjing Port, Jiangsu Province, China, on 9 August 2018. The construction data include 23,265 data sets (rows) with 238 dimensions (columns) from 9:33–15:17 BJT on the studied day.

**Figure 6:**
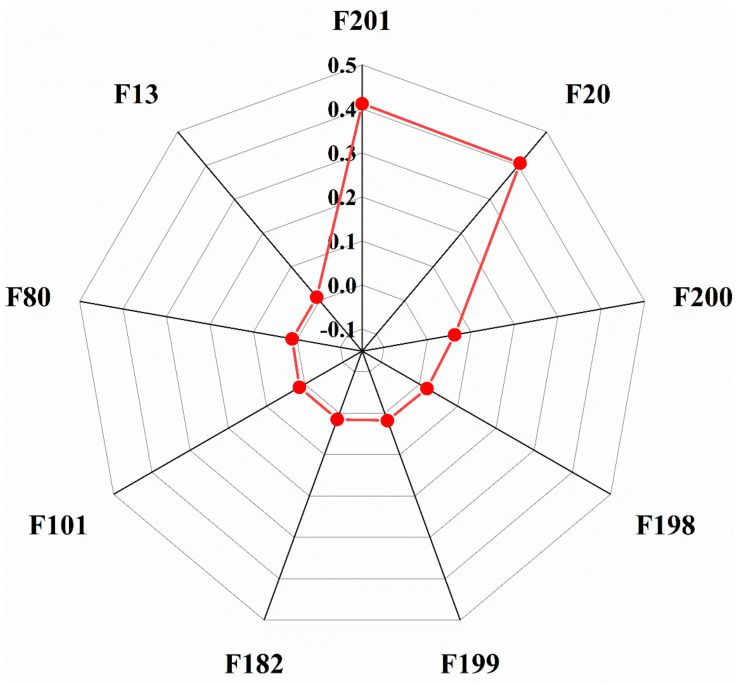
Feature importance ranking.

Similar to case 1, in case 2, first, the original data were preprocessed, then RFE was applied for feature extraction of the data, and 9 feature vectors were selected. Their feature importance is shown in Figure 6.

Where F201 is the vacuum of the underwater dredger pump, F20 is the flow rate, F200 is the #2 main pump discharge pressure, F198 is the discharge pressure of the underwater dredger pump, F199 is the discharge pressure of the #1 main pump, F182 is the carriage stroke, F101 is the #2 main pump rotational speed, F80 is the swing width angle, and F13 is the cutter speed.

The parameters of each model are shown in Table 5.

#### 3.2.2. Evaluation

The evaluation of five base algorithm models as shown in Table 6. Because the construction conditions of case 2 were relatively stable and the data fluctuated little, the $R^{2}$ values of the five basic algorithm models were higher, the RMSE and MAE values were smaller, and the prediction effect was better.

The SG models constructed based on different base models are shown in Table 7. Close inspection of Table 7 reveals that the *R^2^* of SG model 4, with KNN, LightGBM, RF, and XGBoost as the base model and GBDT as the meta model, is the highest among all algorithm models, and MAE and RMSE are also the smallest, indicating that the SG model 4 can accomplish the task of accurately predicting mud concentration.

**Figure 7:**
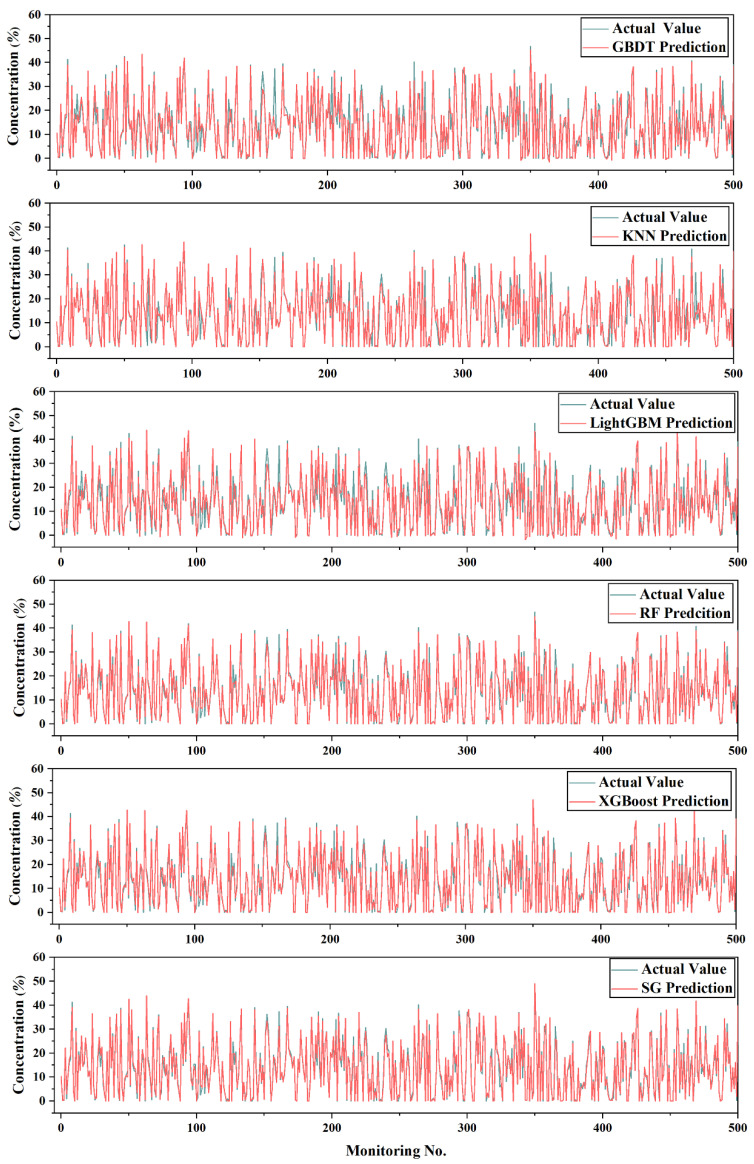
Error comparison.

Therefore, LightGBM, RF, XGBoost, and KNN were used as the base model of the SG model, and the GBDT model was used as the meta-model to obtain the SG algorithm model in case 2. The comparison analysis diagram of the predicted value and the true value of each algorithm model is shown in Figure 7. From the comparison diagram, it can be clearly seen that the error between the predicted value and the true value of the SG model was small and the fit was better.

## 4. Conclusions

This paper proposed a method of soft mud concentration sensors based on data mining. First, the training data set of the target dredger were pre-processed, including removing abnormal data and non-construction data and smoothing and normalizing the data. Second, feature engineering is used to extract features from the processed data, and then the selected features are brought into the selected model for training and learning. Finally, the trained model is applied to actual construction. To improve the accuracy of model prediction, this paper used the stacking method to build a new stacking generalization model for machine learning algorithms such as the GBDT, LightGBM, XGBoost, KNN, and RF methods, which greatly improves the accuracy of model prediction.

The proposed soft sensor method based on the data mining of the mud concentration can realize the real-time online accurate prediction of the mud concentration. In case 1, three of the five SG models have better *R^2^* values than the base model, and all five SG models are better than the base models in case 2, in which conclusion is basically the same as Tyralis [45]. In particular, the goodness of fit of the stacking generalization model in the two actual cases was higher than 0.9, and the prediction effect was significant. 

As an auxiliary supplementary method for the current marine γ-ray concentration meter, the soft sensor method was more accurate than the other auxiliary measurement methods. This method can play an emergency supplementary role when the γ-ray concentration meter fails, and it can also be used alternately with the γ-ray concentration meter under stable working conditions, which greatly extends the service life of the γ-ray concentration meter.

There are two points in this study that require special attention:

(1) Before learning the construction data of the target dredger, special attention should be paid to data preprocessing. The high-quality preprocessing process is extremely important for the accuracy of the prediction.

(2) From the two cases studied, it was found that the three sensor data of the discharge pressure of the main pump, the flow and the suction vacuum of the underwater dredger pump had a prominent influence on the mud concentration, which also verifies the cognition in the actual construction from the data level. To verify the importance of these three features in the prediction of the mud concentration, the SG model was used to train and test these three features in two cases. The result shows that in case 1, the *R^2^* is 0.897, the MAE is 1653, and the RMSE is 2.752, and in case 2, the *R^2^* is 0.941, the MAE is 2.025, and the RMSE is 3.136, which indicates that in some scenarios where the selected features cannot be employed, using only these three features for training prediction can also achieve better results.

The proposed mud concentration soft sensor method is a supplementary method to the current marine γ-ray concentration meter, which temporarily replaces the γ-ray concentration meter when the γ-ray concentration meter fails or when the working conditions are stable. However, it cannot completely replace the γ-ray concentration meter. In the construction process, the soft-sensing model only needs to read the data of the specified characteristic sensor to predict the mud concentration data in real time, which mitigates the problem of a failing mud concentration meter under severe working conditions, as well as an inferior construction operation. This technology temporarily replaces the work of the mud densitometer under stable conditions and then improves the service life of the mud densitometer. Soft-sensing release has advantages of low price, simple operation, high efficiency and reliability, which solves the problem of a single method for detecting the mud concentration of dredging ships. The focus of the next step is to develop a measurement method that can completely replace the γ-ray concentration meter.

## Figures and Tables

**Table 1:** Monitoring data structure of the cutter suction dredger.

ID	F0	F1	F2	F3	F4	F5	…	F238
	Water Level (m)	1 Main Pump Efficiency (%)	Left Trunnion Draft (m)	Right Trunnion Draft (m)	Trunnion Average Draft (m)	Ladder Length (Projection) (m)	…	Main Hydraulic Oil Tank Temperature (°C)
1	2.6863	21.3508	2.7771	2.5987	2.6879	34.5083	…	30.8449
2	1.9	21.3411	2.7641	2.6012	2.6826	34.5103	…	30.8449
3	1.9	21.3347	2.7608	2.6136	2.6872	34.5117	…	30.8449
4	1.9	21.3573	2.7594	2.5871	2.6733	34.5069	…	30.8449
5	1.9	21.3573	2.7594	2.5871	2.6733	34.5069	…	30.8449
…	…	…	…	…	…	…	…	…

**Table 2:** Parameters of the five models.

Model	Parameters
GBDT	eta = 0.05; max_depth = 4; n_estimators = 3000
KNN	n_estimators = 1; leaf_size = 1; *p* = 1
XGBoost	eta = 0.5; max_depth = 5; n_estimators = 2200
RF	n_estimators = 100; max_depth = 100; random_state = 55
LightGBM	eta = 0.05; n_estimators = 720; max_bin = 55; num_leaves = 5

**Table 3:** Evaluation of the single model.

	*R* ^2^	MAE	RMSE
KNN	0.4825	3.481	4.755
LightGBM	0.6887	2.745	3.686
RF	0.9106	1.286	1.975
GBDT	0.9113	1.276	1.968
XGBoost	0.9468	0.966	1.524

**Table 4:** Comparison of the SG model.

	SG Model 1	SG Model 2	SG Model 3	SG Model 4	SG Model 5
KNN	Meta model	Base model	Base model	Base model	Base model
LightGBM	Base model	Meta model	Base model	Base model	Base model
RF	Base model	Base model	Meta model	Base model	Base model
GBDT	Base model	Base model	Base model	Meta model	Base model
XGBoost	Base model	Base model	Base model	Base model	Meta model
*R^2^*	0.9023	0.9549	0.9745	**0.9774**	0.9330
MAE	1.362	0.924	0.626	**0.595**	1.152
RMSE	1.956	1.402	1.048	**0.987**	1.699

**Table 5:** Parameters of the five models.

Model	Parameters
GBDT	eta = 0.05; max_depth = 3; n_estimators = 2000
KNN	n_estimators = 5; leaf_size = 50; *p* = 1
XGBoost	eta = 0.01; max_depth = 5; n_estimators = 2000
RF	n_estimators = 100; max_depth = 50; random_state = 55
LightGBM	eta = 0.1; n_estimators = 720; max_bin = 55; num_leaves = 5

**Table 6:** Evaluation indexes of the four models.

	*R* ^2^	MAE	RMSE
LightGBM	0.9309	1.9911	2.9591
GBDT	0.9367	1.7354	2.8323
KNN	0.9402	1.1952	2.7514
XGBoost	0.9649	1.1782	1.7856
RF	0.9681	1.0974	1.7651

**Table 7:** Comparison of the SG model.

	SG Model 1	SG Model 2	SG Model 3	SG Model 4	SG Model 5
KNN	Meta model	Base model	Base model	Base model	Base model
LightGBM	Base model	Meta model	Base model	Base model	Base model
RF	Base model	Base model	Meta model	Base model	Base model
GBDT	Base model	Base model	Base model	Meta model	Base model
XGBoost	Base model	Base model	Base model	Base model	Meta model
*R* ^2^	0.9691	0.9904	0.9791	**0.9919**	0.9909
MAE	1.1030	0.6802	0.9731	**0.6200**	0.6491
RMSE	1.7963	1.107	1.6245	**1.0205**	1.075
